# Roles of circRNA dysregulation in esophageal squamous cell carcinoma tumor microenvironment

**DOI:** 10.3389/fonc.2023.1153207

**Published:** 2023-06-13

**Authors:** Jingyi Li, Yuxia Song, Huihong Cai, Bo Zhou, Jun Ma

**Affiliations:** ^1^ Department of Clinical Laboratory, The Second Affiliated Hospital of Zhengzhou University, Zhengzhou, Henan, China; ^2^ Department of Reproductive Medicine, The Second Affiliated Hospital of Zhengzhou University, Zhengzhou, Henan, China; ^3^ Medical Research Center, The Second Affiliated Hospital of Zhengzhou University, Zhengzhou, Henan, China

**Keywords:** circRNA, esophageal squamous cell carcinoma, tumor microenvironment, biological mechanism, chemoradiation resistance

## Abstract

Esophageal squamous cell carcinoma (ESCC) is the most prevalent histological esophageal cancer characterized by advanced diagnosis, metastasis, resistance to treatment, and frequent recurrence. In recent years, numerous human disorders such as ESCC, have been linked to abnormal expression of circular RNAs (circRNAs), suggesting that they are fundamental to the intricate system of gene regulation that governs ESCC formation. The tumor microenvironment (TME), referring to the area surrounding the tumor cells, is composed of multiple components, including stromal cells, immune cells, the vascular system, extracellular matrix (ECM), and numerous signaling molecules. In this review, we briefly described the biological purposes and mechanisms of aberrant circRNA expression in the TME of ESCC, including the immune microenvironment, angiogenesis, epithelial-to-mesenchymal transition, hypoxia, metabolism, and radiotherapy resistance. As in-depth research into the processes of circRNAs in the TME of ESCC continues, circRNAs are promising therapeutic targets or delivery systems for cancer therapy and diagnostic and prognostic indicators for ESCC.

## 1 Introduction

As one of the most prevalent malignant tumors, esophageal squamous cell carcinoma (ESCC) is associated with a high mortality rate worldwide (1). In 2020, there were 604,100 new esophageal cancer (EC) cases and 544,076 EC-related deaths, ranking seventh and sixth in cancer morbidity and mortality, respectively (2, 3). ESCC accounts for approximately 85% of all EC cases worldwide (4). Currently, available treatment strategies for ESCC include surgical resection, chemotherapy, radiotherapy, molecular targeted therapy, and their combinations (5). However, due to the high probability of recurrence, early metastasis, and extremely low five-year survival rate, the patient prognosis continues to be poor (6, 7).

CircRNAs have been identified and represent unusual canonical and non-canonical RNA splicing errors (8). However, high-throughput sequencing and specialized computational pipelines have led to recognizing circular RNAs (circRNAs) as a class of endogenous non-coding RNA existing objectively in multiple species without a 5’ end cap and a 3’ end poly(A) tail, forming a circular structure with covalent bonds rather than “transcriptional noise.” (9, 10) Such a unique loop structure makes circRNAs more stable than linear RNAs because they are resistant to RNA exonucleases and RNase R nucleic acid exonucleases (11). CircRNAs have been recently discovered to be involved in cancer growth, metastasis, recurrence, and therapy resistance by regulating sponging miRNAs, binding with proteins, and encoding proteins and peptides (12–16).

The communication between ESCCs and other cells and the interaction among different cell types in the tumor microenvironment (TME) determines ESCC development and progression (6, 17). To establish a TME, tumor cells change their normal developing environment as cancer progresses (18). Through interaction with tumor cells, the TME, constituting the region around the tumor during development, is vital in cancer-related disorders (19). The TME plays a significant role in carcinoma progression, including proliferation, metastasis, immune evasion, and chemoradiation resistance (20). In addition, extracellular metabolites, such as exosomes, which are typically communication signals between various cellular compartments, frequently play a role in the complicated interactions between tumor cells, normal cells, their microenvironment, and the accompanying stroma (18). CircRNAs primarily contribute to and control ESCC development by affecting the TME immunological microenvironment, metabolism, hypoxia, angiogenesis, and epithelial-to-mesenchymal transition (EMT) (19, 21, 22). The development of more potent ESCC therapies may be facilitated by establishing a circRNA-involved TME network, with tailored therapy being an option based on the interaction with circRNAs (23–25).

In this review, we explored the irreplaceable role of circRNAs in regulating cancer progression in the TME of ESCC. In summary, circRNAs exhibit good development potential despite the TME studies on them being in their early stages. CircRNA research translation into clinical applications is required.

## 2 Biogenesis regulation, degradation, and function of circRNA

**Figure 1:**
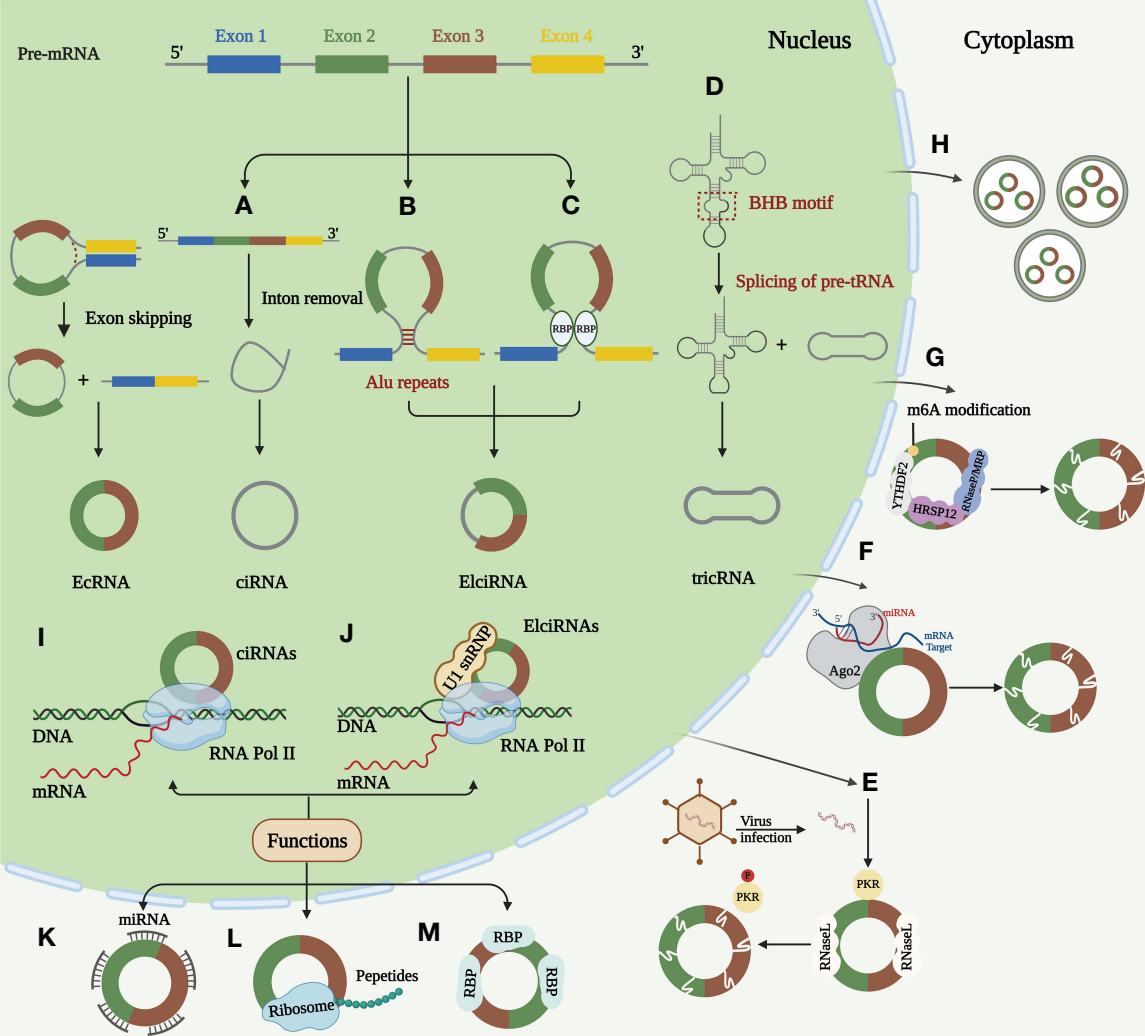
Biogenesis, degradation, and functions of circRNAs. **(A)** Lariat-driven circularization. The skipped exon and intron sequences are spliced from the previous mRNA. The 5 ‘splice receptor and 3’ splice donor are connected to form a lariat structure. Larch further undergoes internal splicing to remove the intron sequence, thus generating EIciRNA or EcRNA. Some spliced introns can be cyclized to form ciRNA. **(B)** Intron paring-driven circularization. This circularization depends on the complementary base pairing of different fan introns to form a hairpin structure. The complementary sequence in the flanking intron of the exon is spliced after splicing, so that the downstream 5 ‘splice site is close to the upstream 3’ splice site for cycle. Most intron pairing patterns are promoted by ALU duplication. **(C)** RBPs-driven circularization. RBP binds to the RBP binding sites on the sector sequence, and these two RBPs can facilitate the cycle by bringing the splicing sites closer together. **(D)** Formation of tricRNAs. Introns carrying pre-tRNA are cut at the characteristic bulge helix bulge (BHB) motif by the tRNA splicing endonuclease (TSEN) complex. Ligase links the RNA that is generated at the end of the tron to form stabilized tricRNA. **(E)** Active RNase L triggers circRNA degradation upon virus infection, which relieves the suppression of PKR. **(F)** miRNAs can bind to circRNAs through base pairing and guide Ago2 dependent cleavage. **(G)** CircRNA containing m6A is recognized by YTHD F2 which can interact with RNase P/MRP bridged by HRSP12 and then the complex initiating the nuclear degradation of circRNAs. **(H)** CircRNAs can be excreted to the extracellular space by exosomes. Function of circRNAs. **(I)** Transcriptional regulation. ciRNAs regulates the transcription of host genes by promoting the extension activity of RNA Pol II. **(J)** Transcriptional regulation. ElciRNAs can combine with U1 snRNP and then interact with RNA Pol II to regulate host gene transcription in nucleus. **(K)** miRNA sponging. CircRNAs act as miRNA sponge to regulate the function of miRNA target mRNAs. **(L)** Coding of proteins and peptides. CircRNAs can perform regulatory functions by encoding proteins and peptides. **(M)** RNA binding proteins. CircRNAs can combine with RBPs by different mechanisms.

For a long time, most circRNAs were considered “splicing noise” or “byproducts of RNA processing.” (26) In recent years, circRNAs have been increasingly studied and observed to play essential roles in normal cell differentiation, tissue homeostasis, and disease development. Their expression is usually independent of host gene expression (27). CircRNAs are stable byproducts of mRNA splicing and products of newly regulated selective splicing, which have distinctive molecular mechanisms (28). This circular structure endows circRNAs with many distinct features: sequence conservation, evolutionary species conservation, tissue-specific expression patterns, and high abundance and stability, demonstrating that circRNAs have vital non-coding functions (29–31). We will focus later on the regulation, degradation and function of circRNA (**Figure 1**).

### 2.1 Biogenesis, regulation, and degradation of circRNA

Most circRNAs are produced by reverse splicing of precursor mRNAs (pre-mRNAs), creating a single-stranded covalent closed-loop structure by connecting the upstream 3′ splice acceptor site to the downstream 5′ splice donor site containing a specific junction site. This is followed by removing all or part of the intron by the spliceosome and ligating the remaining sequences, producing different circRNAs (32, 33). This procedure was performed under the regulation of cis-acting elements and trans-acting factors (34). CircRNAs can be divided into three main categories according to their source sequences: exonic circRNAs (EcRNAs), intronic circRNAs (ciRNAs), and exon-intron circRNAs (EIciRNA) (35–37). The back-splicing hypothesis models currently proposed to describe circRNA generation are Lariat-driven circularization, intron pairing-driven circularization, and RNA-binding protein (RBP)-driven circularization. The three models have different mechanisms (11, 28, 32).

Lariat-driven circularization occurs while removing introns or exons from pre-mRNAs (38, 39). Intron pairing-driven circularization: The basis of this type of circulation is the complementary base pairing of various fan introns, and complementary sequences—short repeat elements (e.g., ALU repeats) or non-repeat elements—in introns flanking post-snap exons are important cis-acting elements of circRNA biogenesis (40). Regarding RBP-driven circularization, fan sequences contain RBP binding sites. By bringing the splice sites close, both RBPs can enhance recycling (41). Certain RBPs are trans-acting regulators of circRNA synthesis (42). In contrast, some RBPs may prevent circRNA synthesis by preventing proper matching of intron elements (43, 44). In addition to pre-mRNA production, a minor portion of intron-derived circRNAs is formed by precursor tRNA (pre-tRNA) splicing (45). RNA synthesized at the intron end is ligated by ligase into a stable circRNA called tRNA intronic circular RNA (tricRNA) (45, 46).

The high circRNA expression in cancer is often closely associated with poor prognosis. Therefore, understanding circRNA degradation and inactivation mechanisms is crucial for emerging targeted therapies (12). A study revealed that circRNAs can be degraded by RNase L. Endogenous circRNAs frequently form defective double complexes, preventing protein kinase (PKR) activation by double-stranded RNA (dsRNA)-activated PKR. Their reduction triggers abnormal PKR activation and autoimmune responses (47). Argonaute 2 (Ago2)-mediated RNA degradation is the most commonly reported mode of circRNA degradation (48, 49). Ago2 is a nucleic acid endonuclease that uses endogenous guide RNAs, such as miRNAs (50). However, Ago2-dependent circRNA degradation also has some drawbacks, as it is inapplicable to circRNAs without specific miRNA targets (51). The most frequent RNA modification is m6A (52). Recent studies have revealed that YTHDF2 recognizes circRNAs containing m6A, and HRSP12 links YTHDF2 and RNase P or MRP, enabling RNase P/MRP to initiate the intranuclear degradation of YTHDF 2-bound circRNAs (53). According to recent research, all circRNAs that possess an open reading frame (ORF) were predicted to possess m6A sites (54). Besides intracellular pathways, circRNAs can be further exported to extracellular vesicles contributing to their clearance (55). Cancer progression caused by circRNA dysregulation promises new clinical therapeutic advances as the biogenesis and degradation mechanisms of circRNAs are further investigated.

### 2.2 Biological functions of circRNAs

The biological roles of circRNAs have also been thoroughly explained. According to recent studies, circRNAs perform their tasks using four primary methods: miRNA sponging, transcription regulation, protein and peptide coding, and RNA-binding protein incorporation (27, 28, 56). CircRNAs can function as miRNA sponges: Most circRNAs in this review can affect the ESCC TME by acting as miRNA sponges to regulate downstream targets. Moreso, circRNAs act as competing endogenous RNAs (ceRNAs) and microRNA (miRNA) sponges by binding to target mRNAs in a base-paired pattern, causing mRNA cleavage or limiting mRNA translation to regulate miRNA activity on other target genes (10, 56, 57). According to previous reports, circRNAs simultaneously regulate several miRNAs and have multiple miRNA-binding sites. These evolutionarily conserved binding sites guarantee the target efficacy (58). Notably, only a few circRNAs have multiple miRNA-binding sites (59). For instance, Sun et al. have found that a total nine miRNAs and nine candidate mRNA were predicted to have an interaction with has_circ_0000520 (60). CircRNAs regulate transcription: In addition, circRNAs play a significant role in post-transcriptional or transcriptional regulation of gene expression (61, 62). This role is mainly mediated by EIciRNAs and ciRNAs expressed in the nucleus during parental gene regulation (63, 64). The promoter region of the parental gene is bound by a complex formed by specific RNA-RNA interactions between EIciRNA, U1 small nuclear ribonucleoprotein (U1snRNP), and U1 small ribonucleic acid (snRNA). This complex then interacts with RNA Pol II to enhance and promote the transcription of the parental gene (63, 65). ciRNAs regulate their parental function by boosting RNA Pol II elongation (66). CircRNAs can encode proteins and peptides: Despite circRNAs being non-coding RNAs, they also regulate by encoding proteins and polypeptides (67–69). Studies have revealed a connection between circRNAs and polymers, a portion containing the initiation codon AUG and a putative ORF of favorable length, indicating that circRNAs have protein-coding potential (70, 71). Although circRNAs are classified as lncRNAs, they can encode some short peptides. The coding circRNA contains an internal ribosomal entry site (IRES) that allows translation of some of the coding sequences in the circRNA (72). Proteomics application has provided the most visual evidence that ncRNAs can encode regulatory peptides (71). Pamudurti et al. demonstrated that circMbl is capable of producing proteins of approximately 10kDa size in Drosophila by establishing intron-exon-intron minigenes (73). Studies have revealed that some proteins or peptides encoded by circRNAs play irreplaceable regulatory roles in tumorigenesis and progression (74–78). CircRNAs can interact with RNA-binding proteins: In addition to the functions mentioned above, circRNAs combine with RBPs through different mechanisms. RBPs are a broad category of proteins involved in the transcription and translocation of genes and can serve as fundamental elements of circRNA function (41). Three primary modes of action have been described for circRNA binding to RBPs: formation of protein complexes (79), inhibition of protein function (protein decoys) (80), and interactions between different proteins (81). The first is the formation of protein complexes: circRNAs with multiple protein-binding sites can serve as dynamic scaffolding for assembling massive RNA-protein complexes by modulating protein-protein interactions (82). The second category is protein decoys, where circRNA-encoded proteins are in competing with their homologous linear splice protein isoforms for binding molecules, thereby inhibiting normal isoform function (80). Finally, circRNAs bind and chelate specific proteins and regulate certain protein-protein and protein-RNA interactions (81, 83). CircRNAs can form distinct circRNA-protein complexes (circRNPs) by interacting with various proteins, changing the related proteins’ mode.

## 3 CircRNA dysregulation modulates the clinical characteristics and biological processes of ESCC

A few aberrantly produced circRNAs were recently revealed to control the proliferation, invasion, migration, and apoptosis of ESCC in TME, thereby affecting ESCC progression. This could lead to new insights into circRNAs as therapeutics. Here, we summarize the biological functions and clinicopathological features of the aberrantly expressed circRNAs in ESCC (**Table 1**).

**Table 1:** The clinical and cytological functions of circRNAs in ESCC.

CircRNA	Expression in ESCC	Clinical correlation	Cell function	Refs
Circ_0048117	upregulated	invasion depth, lymph node metastasis, distant metastasis and TNM stage (predicted an advanced T and N stage)	proliferation, migration, invasion, metastasis, apoptosis	(84)
CircTCFL5	upregulated	tumor growth, tumor volume	proliferation, migration, invasion, apoptosis	(85)
CircGOT1	upregulated	tumor size, tumor weight, overall survival rate, prognosis	proliferation, migration	(86)
CircFNDC3B	upregulated	tumor growth, TNM stage, lymph node invasion, clinical stage III	proliferation, migration, invasion, metastasis, apoptosis	(87)
Circ_0001093	upregulated	lymph node metastasis, TNM stage, tumor size	proliferation, migration, invasion	(88)
CircOGDH	upregulated	tumor growth	proliferation, metastasis, invasion	(89)
Circ_0000705	upregulated	lymph node metastasis, TNM stage, prognosis	proliferation, invasion, migration	(90)
CircPUM1	upregulated	tumors size, tumor volume, tumor weight	proliferation, colony formation, pyroptosis	(91)
Circ_0072088	upregulated	tumor size, invasion depth, TNM stage, and LNM	proliferation, migration, and invasion	(92)
CircDUSP16	upregulated	tumors growth, tumor volume, tumor weight	cell viability, colony formation, migration, invasion	(93)
Circ_0007624	downregulated	tumor growth, overall survival rate	proliferation, migration, invasion, apoptosis	(94)
CircHIPK3	upregulated	tumor size, tumor differentiation, lymph node metastases	proliferation, migration	(95)
CircDOCK5	downregulated	overall survival rate, prognosis	metastasis, migration, invasion	(96)
CircNTRK2	upregulated	TNM stage, lymph node metastasis, overall survival rate	proliferation, invasion, apoptosis	(97)
Hsa_circ_0012563	upregulated	overall survival rate	migration, invasion, emt, apoptosis, G1 arrest	(98)
Hsa_circ_0006948	upregulated	overall survival rate, lymphatic metastasis, prognosis	proliferation, migration and invasion	(99)
Circ_2646	upregulated	TNM stage (especially N stage), lymph node metastasis, tumor differentiation, pathologic stage	proliferation, migration, invasion	(100)
Hsa_circ_0000277	upregulated	TNM stage, lymphatic metastasis, lymph node metastasis, histological grade	proliferation, invasion	(101)
CircARAP2	upregulated	tumor growth, tumor volume, tumor weight	proliferation, colony formation, metastasis, invasion, cancer stem cell differentiation	(102)
CircLONP2	upregulated	overall survival, disease-free survival	proliferation, migration	(103)
CircFAM120B	downregulated	tumor size, tumor volume	proliferation, migration, invasion, colony number	(104)
CircVRK1	downregulated	overall survival rate, prognosis	proliferation, migration, invasion	(105)
Circ_0007022	upregulated	overall survival rate, tumor growth, tumor weight	proliferation, colony formation, metastasis, migration, invasion, apoptosis	(106)
Circ_100367	upregulated	overall survival rate, tumor growth, tumor weight	proliferation, colony formation, metastasis, migration, invasion, apoptosis	(107)
Hsa_circ_0014879	upregulated	tumor growth, tumor volume, tumor weight	proliferation, colony formation, metastasis, migration, invasion, apoptosis	(108)
CircMAN1A2	upregulated	overall survival rate, disease-free survival, tumor growth, tumor volume, tumor weight	proliferation, colony formation	(109)
Hsa_circ_0007142	upregulated	tumor volume, tumor weight	proliferation, migration, apoptosis	(110)
CircDOPEY2	downregulated	overall survival rate, progression-free-survival, tumor volume	proliferation, colony formation, apoptosis	(111)
Hsa_circ_0000277	upregulated	tumor stage, tumor growth, lymph node metastasis, prognosis	proliferation, colony formation, apoptosis, cell cycle	(112)
Circ_0006168	upregulated	tumor growth, tumor volume, tumor weight	proliferation, colony formation, migration, invasion, apoptosis	(113)
CircPVT1	upregulated	tumor growth, tumor volume, tumor weight	proliferation, colony formation, migration, invasion, apoptosis	(114)
Hsa_circ_ 0026611	upregulated	TNM stage, lymph node metastasis, poor prognosis, OS, DFS	/	(115)
Circ_0000337	upregulated	tumor growth, tumor volume, tumor weight	proliferation, colony number, migration, invasion, apoptosis	(116)
CircSFMBT2	upregulated	overall survival rate, tumor volume, tumor weight	proliferation, colony number, invasion, apoptosis	(117)

ESCC mainly carries out distant metastasis through the lymphatic system, causing patients to lose the opportunity for early surgery, and is directly related to poor prognosis (118–120). High expression of circ_0048117, circ_0001093, circ_0000705, and circHIPK3 are closely related to lymph node metastasis and the TNM stage of ESCC (84, 88, 90, 95). Conversely, as a tumor suppressor, low circDOCK5 expression was positively correlated with overall survival (96). In addition, the highly expressed circNTRK2, hsa_circ_0006948, circ_2646, and hsa_circ_0000277 were closely associated with lymph node metastasis and overall survival time and tumor differentiation (97, 99–101). Cheng et al. revealed that upregulated hsa_circ_0000277 is associated with lymph node metastasis and recurrence in patients with ESCC (112). Liu et al. demonstrated that hsa_circ_0026611 was in the serum of patients with ESCC in the form of serum exosomes and could identify whether ESCC was complicated by lymph node metastasis. In addition, hsa_circ_0026611 was closely related to TNM staging. Thus, hsa_circ_0026611 could be a biomarker in clinical practice (115).

The TNM stage of patients with ESCC is directly related to prognosis and is closely associated with surgery and chemotherapy (121). CircFNDC3B and circ_0072088 are highly expressed in ESCC and are positively correlated with the TNM stage and poor survival (87, 92). In addition, circRNAs are related to tumor volume and weight (85, 86, 89, 91). CircDUSP16, circARAP2, circFAM120B, and hsa_circ_0014879 promote an increased tumor volume by promoting tumor cell proliferation and inhibiting apoptosis (93, 102, 104, 108). Circ_0007142 was upregulated in ESCC tissues and cells and correlated with cisplatin resistance. Mechanistically, circ0007142 increased cell survival by increasing the resistance of tumor cells to cisplatin, leading to increased tumor volume and weight (110). Circ_0006168 resists chemotherapy by reducing paclitaxel sensitivity in resistant cells, increasing tumor volume (113). CircPVT1 is vital in maintaining ESCC chemoresistance to 5-FU through ferroptosis and the Wnt/β pathway. *In vivo*, circPVT1 reduced the sensitivity of tumor cells to 5-FU and increased the tumor volume under chemotherapy (114). As an oncogenic factor, circ_0000337 knockdown can improve the sensitivity of ESCCs to cisplatin, thereby reducing tumor volume and weight *in vivo (* 116).

## 4 CircRNAs as a novel regulator of tumor microenvironment in ESCC

**Figure 2:**
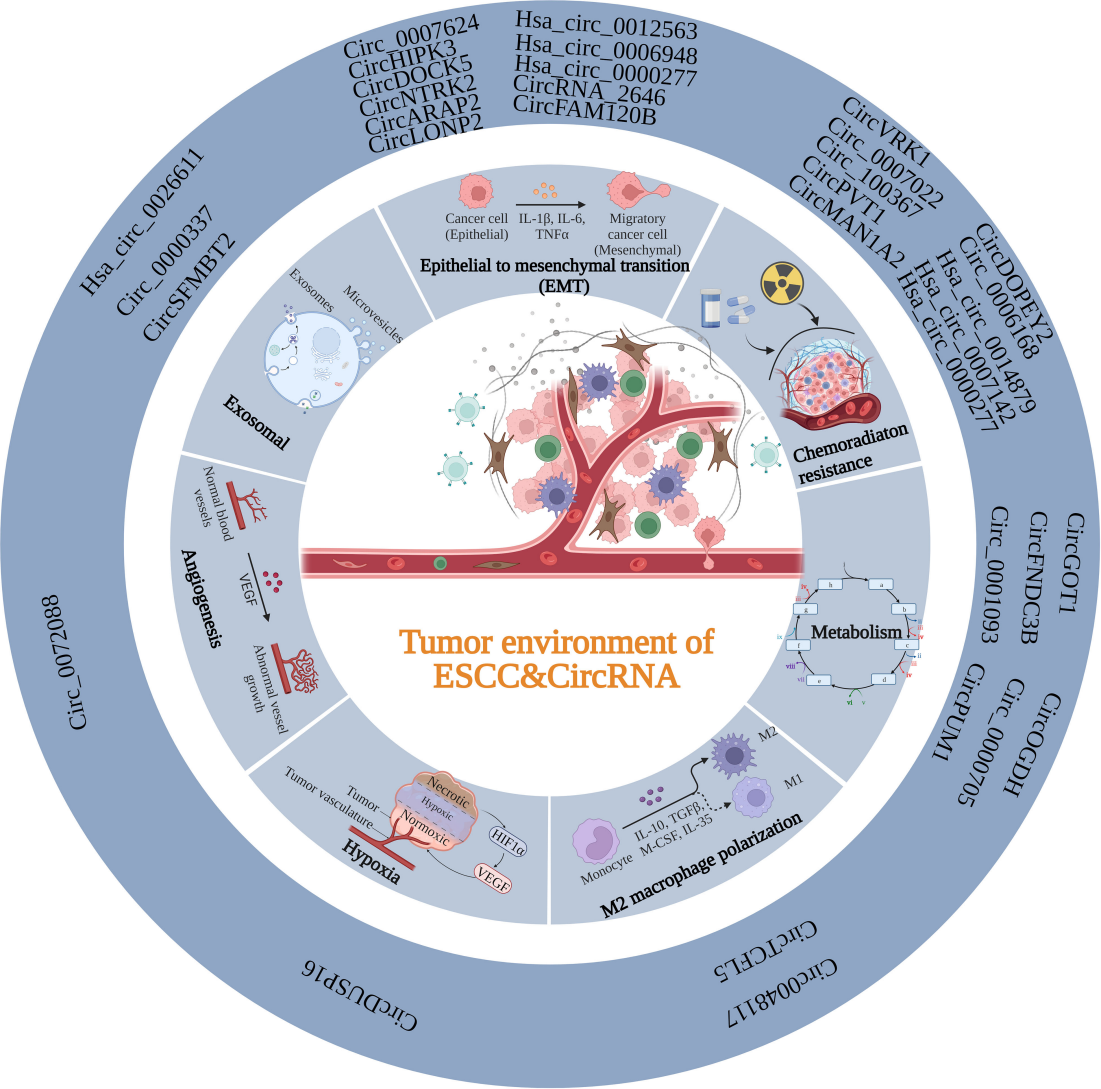
Summary of the function of circRNAs in the TME of ESCC. CircRNAs can participate in the TME of ESCC through various mechanisms, including EMT, exosomal, angiogenesis, hypoxia, M2 polarization, metabolism and chemoradiation resistance.

The TME is a vital factor in determining cancer progression at all stages and is a complex ecosystem in which cancer cells and the stroma around them coexist (122). Recruitment and conversion of tumor cells into nearby normal cells are essential for cancer advancement (123). The TME consists of stromal cells, immune cells, the vascular system, and the extracellular matrix (ECM). Crosstalk among TME components is essential for influencing cancer progression (124–126). As mentioned above, abnormal expression circRNAs levels in ESCC are caused by various mechanisms. The functional mechanisms of circRNAs in ESCC TME are discussed in the following sections. (**Figure 2**; **Table 2**).

**Table 2:** Role of circRNAs in the tumor microenvironment of ESCC.

Biology activities in TME	CircRNA	Property	Molecular axis	Putative Function	Refs
M2 Macrophage Polarization	Circ_0048117	promotor	Circ_0048117/miR-140/toll-like receptor 4 (TLR4)	miRNA sponge	(84)
M2 Macrophage Polarization	CircTCFL5	promotor	CircTCFL5/miR-543- FMNL2	miRNA sponge	(85)
aerobic glycolysis, resistance to cisplatin, EMT	CircGOT1	promotor	CircGOT1/miR-606/GOT1	miRNA sponge	(86)
glycolysis, exosomal	CircFNDC3B	promotor	CircFNDC3B/miR-490-5p/TXNRD1	miRNA sponge	(87)
glutamine metabolism	Circ_0001093	promotor	Circ_0001093/miR-579-3p/GLS	miRNA sponge	(88)
glutamine metabolism	CircOGDH	promotor	CircOGDH/miR-615-5p/PDX1	miRNA sponge	(89)
proline metabolism	Circ_0000705	promotor	Circ_0000705/miR-621/PYCR1	miRNA sponge	(90)
oxidative phosphorylation, hypoxia	CircPUM1	promotor	CircPUM1/UQCRC2	RNA-binding protein-formation of protein complexes	(91)
angiogenesis	Circ_0072088	promotor	Circ_0072088/miR-377/VEGF	miRNA sponge	(92)
hypoxia	CircDUSP16	promotor	CircDUSP16/miR-497-5p/TKTL1	miRNA sponge	(93)
EMT	Circ_0007624	suppressor	Circ_0007624/miR-224-5p/CPEB3/EGFR/PI3K/AKT	miRNA sponge	(94)
EMT	CircHIPK3	promotor	CircHIPK3/miR-124/AKT3	miRNA sponge	(95)
EMT	CircDOCK5	suppressor	CircDOCK5/miR-627-3p/TGF-β/SMAD/ZEB1	miRNA “reservoir”	(96)
EMT	CircNTRK2	promotor	CircNTRK2/miR-140-3p/NRIP1	miRNA sponge	(97)
EMT	Hsa_circ_0012563	promotor	Hsa_circ_0012563/XRCC1/EMT	Coding of proteins and peptides	(98)
EMT	Hsa_circ_0006948	promotor	Hsa_circ_0006948/miR-490-3p/HMGA2	miRNA sponge	(99)
EMT	Circ_2646	promotor	Circ_2646/miR-124/PLP2	miRNA sponge	(100)
EMT	Hsa_circ_0000277	promotor	Hsa_circ_0000277/miR-4766-5p/LAMA1	miRNA sponge	(101)
EMT	CircARAP2	promotor	CircARAP2/miR-761/FOXM1	miRNA sponge	(102)
EMT	CircLONP2	promotor	CircLONP2/miR-27b-3p/ZEB1	miRNA sponge	(103)
EMT	CircFAM120B	suppressor	CircFAM120B/miR-661/PPM1L axis and CircFAM120B/PKR/p38 MAPK/EMT pathway	miRNA sponge/RNA-binding protein	(104)
resistance to radiation, EMT	CircVRK1	suppressor	CircVRK1/miR-624-3p/PTEN/PI3K/AKT	miRNA sponge	(105)
resistance to radiation, EMT	Circ_0007022	promotor	Circ_0007022/miR-338-3p/NRP1/PI3K/AKT	miRNA sponge	(106)
resistance to radiation, EMT	Circ_100367	promotor	Circ_100367/miR-217/Wnt3	miRNA sponge	(107)
resistance to radiation, EMT	Hsa_circ_0014879	promotor	Hsa_circ_0014879/miR-519-3p/CDC25A	miRNA sponge	(108)
resistance to cisplatin	CircMAN1A2	promotor	HNRNPUL1/CircMAN1A2	RNA-binding protein	(109)
resistance to cisplatin	Hsa_circ_0007142	promotor	Hsa_circ_0007142/miR-494-3p/LASP1	miRNA sponge	(110)
resistance to cisplatin	CircDOPEY2	suppressor	CircDOPEY2/CPEB4/TRIM25/Mcl-1	RNA-binding protein-protein scaffold	(111)
resistance to cisplatin	Hsa_circ_0000277	promotor	Hsa_circ_0000277/miR-873-5p/SOX4/ Wnt/β-catenin	miRNA sponge	(112)
resistance to Taxol	Circ_0006168	promotor	Circ_0006168/miR-194-5p/JMJD1C	miRNA sponge	(113)
resistance to 5-FU	CircPVT1	promotor	CircPVT1/miR-30a-5p/FZD3/Wnt/ miR-30a-5p/β-catenin	miRNA sponge	(114)
exosomal	Hsa_circ_ 0026611	promotor	/	/	(115)
exosomal	Circ_0000337	promotor	Circ_0000337/miR-377-3p/JaK2	miRNA sponge	(116)
exosomal	CircSFMBT2	promotor	CircSFMBT2/miR-107/SLC1A5	miRNA sponge	(117)

### 4.1 Modulating the immune microenvironment in ESCC

The term “macrophage polarization” describes the macrophage activation state at a specific moment (127). Activated macrophages are in two groups based on the guidance of various microenvironmental signals: classically activated macrophages (M1) and alternatively activated macrophages (M2) (128). The polarization of M1/M2 macrophages is critical for tumor progression. Historically, M1 macrophages have been thought to have antitumor activities, whereas M2-polarized macrophages, also known as tumor-associated macrophages (TAMs), have anti-inflammatory and pro-tumor actions (129, 130). In contrast to M1’s antitumor properties, M2-polarized macrophages are incredibly adaptable and multifunctional cells, which also significantly contribute to many pro-tumorigenic outcomes through tumorigenesis, together with angiogenic and lymphangiogenic regulation, immune suppression, hypoxia induction, and stemness (131). CircRNAs are generally associated with M2 polarization (132–134); however, the specific mechanism between circRNAs and M2 polarization is unclear. In recent studies, Lu et al. observed that hypoxia encouraged ESCC cells to produce more exosomes rich in hsa_circ_0048117, which might induce TAMs to differentiate into the M2 type via the miR-140/toll-like receptor 4 (TLR4) pathway. Thus, hsa_circ_0048117 can be seen as a macrophage miR-140 “sponge” which directly competes with TLR4 to bind miRNA. In addition, the ability of ESCC cells to invade and migrate may be enhanced by M2 macrophages (84). M2 macrophages can release Arg1, IL-10, and TGF-α which boost the tumor cells’ capacity for invasion and migration and aid in ESCC metastasis. Compared to healthy participants, serum exosomal hsa_circ_0048117 levels were considerably higher in patients with ESCC. Higher exosomal levels of circ_0048117 were strongly linked to TNM grade and predicted an advanced T and N stage (84). Similarly, in another research, Lin et al. discovered that circTCFL5 was markedly increased in ESCC and could speed up the disease evolution by upregulating FMNL2 by sponging miR-543 *in vitro* and *in vivo*. Mechanistically, circTCFL5 accelerates esophageal cancer development by modifying M2 macrophage polarization through the miR-543/FMNL2 axis (85).

Previous studies have revealed that circRNAs can interact with the tumor immune microenvironment by participating in multiple approaches (19). In addition to participating in M2 polarization and interacting with macrophages, circRNAs mediate tumor immune surveillance (135, 136), regulate immune escape via PD-L1 (137–140), regulate natural killer cells’ cytotoxicity (141–143), and regulate neutrophils, myeloid-derived suppressor cells, and cancer-associated fibroblast (144). However, whether circRNA affects ESCC TME through the aforementioned effects, except M2 polarization, has not yet been reported.

### 4.2 Energy metabolism regulation in the TME

Tumors caused by genomic instability frequently exhibit energy metabolism reprogramming (30). Deregulation of metabolism, common in tumor development, is a crucial source of growth and division. Proteins and non-coding RNAs (ncRNAs) are involved in orchestrating these changes in energy metabolism (145). CircRNAs, a novel class of regulatory molecules, regulate cancer metabolism. CircRNAs regulate the energy metabolism of ESCC through different targets.

Glucose is a vital carbon substrate for generating energy and metabolic intermediates (146). Cancer cells frequently rely on glycolytic metabolism for maintenance; nonetheless, cellular reprogramming of metabolic processes frequently occurs in developing tumors when blood perfusion is constrained (147). For instance, circGOT1 functions as an oncogene in ESCC, which promotes aerobic glycolysis through the miR-606/GOT1 axis, acting as an mRNA sponge (86). CircGOT1 knockdown reduces ESCC cell motility, proliferation, and chemoresistance to cisplatin (86). Additionally, circGOT1 inhibition decreased lactate synthesis, glucose consumption, and ATP levels (86). Further, circFNDC3B was upregulated in ESCC tissues and cells, and circFNDC3B knockdown caused apoptosis, decreased cell glycolysis, and inhibited xenograft tumor formation (87). Sponging miR-490-5p and upregulating TXNRD1 expression resulted in the aforementioned inhibitory effects (87). TXNRD1 overexpression reduced the repressive function by enhancing cell proliferation, migration, and glycolysis and lowering apoptosis in ESCC (87). In addition, tumors from patients with clinical stage III tumors expressed more circFNDC3B than tissues from individuals with clinical stage I + II (87).

Amino acid metabolism is involved in cancer progression. Cancer cells exert worsened metabolic activity to maintain their increased proliferation and microenvironmental adaptation, to survive in nutrient-poor environments. Tumors exhibit increased energy-producing processes such as glycolysis, glutaminolysis, and fatty acid production (148). Since the 1950s, researchers have discovered that glutamine is the amino acid most tumor cells use. The fundamental biological activities require glutamine, which glutaminase transforms into glutamate. Glutamate can also provide nitrogen for purines and pyrimidines production or participate in the TCA cycle via conversion to α-ketoglutarate (145). Glutamate is another source of glutathione, a vital cellular antioxidant. Glutamine’s catabolism creates an anaplerotic pathway that feeds the Krebs cycle; therefore, some cancer cells depend highly on it (149). Thus, glutamine is crucial for tumor development. Recently, Qian et al. discovered that circ_0001093 is overexpressed in ESCC and functions as an mRNA sponge for miR-579-3p to promote GLS expression, boosting glutamine metabolism and malignant phenotype of ESCC (88). Similarly, miR-615-5p was sponged by circOGDH to release PDX1, which increased glutamine metabolism and promoted tumor progression in ESCC (89). Further studies have revealed that elevated miR-615-5p reduces ATP content, α-KG synthesis, and glutamine consumption, which are suppressed by PDX1 (89).

In addition to glutamate metabolism, circRNAs influence ESCC progression by participating in other amino acid metabolism pathways. Proline metabolism is essential to metabolic reprogramming in some malignancies, producing ATP. In cancerous cells, proline metabolism is associated with ATP synthesis, protein and nucleotide production, and redox balance (150–153). Recent studies have demonstrated that proline metabolism is critical for metabolic reprogramming, promoting cell proliferation, preventing cell apoptosis, and generating metabolites that support cancer cell survival in various stressful environments (153–157). Circ_0000705 knockdown boosted ROS generation, decreasing proline and PYCR1 expression and ATP synthesis in ESCC cells (90). Additionally, the inhibitory effects of circ_0000705 knockdown on the aforementioned biological functions in ESCC were reversed by miR-621 suppression or PYCR1 expression restoration (90). Therefore, circ_0000705 might accelerate ESCC development by targeting the miR621/PYCR1 axis and promoting proline metabolism (90).

In addition, increased oxidative phosphorylation may contribute to hypoxia in cancerous cells; however, the exact mechanism underlying this phenomenon is currently being investigated. There are few reports on the potential role of circRNAs in the oxidative phosphorylation of tumor cells (158–160). CircPUM1 is essential for maintaining mitochondrial complex III integrity (91). In ESCC, circPUM1 serves as a scaffold for UQCRC1 binding to UQCRC2 (91). Lower intracellular oxygen levels, significantly suppressed oxidative phosphorylation, decreased mitochondrial membrane potential, and increased ROS production are effects of circPUM1 knockdown (91). Mitochondrial complex III becomes dysfunctional because of circPUM1 deficiency (91). In addition, circPUM1 disruption causes pyroptosis, which initiates ESCC cell death by altering the intracellular ATP levels (91).

Several circRNAs are tightly linked to glycolysis, glutamine metabolism, proline metabolism, and oxidative phosphorylation. In addition, circRNAs may affect ESCC progression through other mechanisms, such as serine and lipid metabolism (145). We could uncover the mystery of ESCC development with further studies in this area.

### 4.3 Abnormal regulation of angiogenesis

Angiogenesis is a crucial process in growth and development and has been the focus of research in recent years. Endothelial cells (ECs) proliferate, differentiate, and migrate during angiogenesis to form new blood vessels based on pre-existing capillaries or venules (161, 162). Additionally, angiogenesis mainly involves cell migration, proliferation, and vascular endothelial growth factor (VEGF) (163–165). A characteristic of cancer is angiogenesis induction (166). The significance of malignant cell neovasculature in tumor biology and nutrient and oxygen delivery to growing tumors is crucial, including tumor metastasis/dissemination (167), metabolic dysfunction (168), and cancer stem cell maintenance (169, 170). CircRNAs within the TME can influence various physiological and pathological processes, including tumor angiogenesis (171, 172). Circ_0072088 is involved in tumor biological processes, such as proliferation, migration, invasion, and apoptosis in different cancers (173–179). Fang et al. verified that circ_0072088 upregulation considerably enhanced the protein and mRNA expression of VEGF in ESCC by sponging miR-377 (92). According to previous studies, VEGF upregulation by circ_0072088 can be reversed by overexpressing miRNA-377 (92). Thus, circ_0072088 can lessen its inhibitory effect on VEGF expression by modulating the miR-377/VEGF axis (92). This could increase angiogenesis regulation, which would encourage ESCC progression.

### 4.4 Regulation of hypoxia in the TME

Oxygen homeostasis is crucial in human physiology and metabolism. Oxygen is required by the machinery for energy production and by many cofactors and substrates for enzymes (180). Hypoxia is a vital component of physiological and pathological processes. Understanding the mechanisms underlying angiogenesis, glucose metabolism, and cell proliferation is particularly important (181). Hypoxia is a typical TME characteristic of various malignancies. The rapid proliferation of cancer cells does not match a lack of oxygen. Thus, several adaptive cellular responses and biological changes are initiated when cancer cells sense a drop in oxygen levels, causing them to swiftly adapt to the hypoxic environment and accelerate cancer progression (182). Recent studies have revealed that circRNAs play crucial roles in developing hypoxic microenvironments and exhibit variable expression in response to hypoxia (183, 184). According to previous studies, miR-497-5p is targeted by PRKAA1 and contributes to ESCC cell proliferation and motility control by being minimally expressed in ESCC (185). In addition, low miR-497-5p expression regulates the radiosensitivity of ESCC by directly targeting the 3’-UTR of CDC25A (186). However, Ma et al. illustrated that miR-497-5p overexpression, which is sponged by circDUSP16, could inhibit the ESCC cell progression under hypoxic conditions (93). In hypoxic conditions, ESCC cells had higher levels of circDUSP16 (93). By inhibiting miR-497-5p and increasing TKTL1, circDUSP16 knockdown restored hypoxia-stimulated malignant tendencies in ESCC cells (93). Under hypoxic conditions, circDUSP16 knockdown clearly hindered ESCC survival, colony formation, and invasion (93). Furthermore, the reduction in glucose intake, lactate generation, and HK2 and LDHA levels following circDUSP16 knockdown demonstrated that circDUSP16 silencing caused an apparent inhibition of glycolysis in ESCCs under hypoxic conditions (93). Additionally, circDUSP16 knockdown prevents ESCC carcinogenesis *in vivo (* 93). These findings suggest that circDUSP16 knockdown partially alters the biologically malignant responses of ESCCs to hypoxia (93).

Hypoxia significantly influences tumor progression, metastasis, invasion, angiogenesis, epigenetic reprogramming, metabolic reprogramming, immune evasion, and glycolysis in ESCC (187–190). Further proof of the possible applications of circRNAs may be provided by understanding the mechanisms of circRNAs in hypoxia.

### 4.5 Inducing epithelial-to-mesenchymal transition and tumor cell migration

The complex program of cell plasticity, EMT, is abnormally reactivated in cancer. The interaction between cancerous cells and TME is crucial for EMT and tumorigenesis (191). CircRNA-related signaling pathways, such as the PI3K/Akt and TGF- signaling axes, are involved in the EMT process. For instance, the PI3K/Akt pathway is affected by circ_0007624 (94). This circRNA functions as a ceRNA to sponge miR-224-5p, increasing CPEB3 expression and inactivating the EGFR/PI3K/AKT axis, which could prevent cell proliferation, metastasis, and EMT in ESCC (94). CircHIPK3 is another circRNA that directly modulates AKT3 expression, acting as an miR-124 sponge in regulating ESCC progression (95). Vimentin and E-cadherin expression were drastically reduced by circHIPK3 expression, indicating that circHIPK3 prevented EMT (95). CircDOCK5 acts as a miR-627-3p reservoir in the TGF-β signaling pathway by stabilizing miR-627-3p to impede TGFB2 and repressing secreted TGF-β, which then dysregulates ZEB1 expression and inhibits EMT (96). In other words, circDOCK5 prevents EMT in ESCC by affecting the miR-627-3p/TGF-β/SMAD/ZEB1 pathway (96).

Despite this, emerging research has revealed that circRNAs are involved in EMT regulation and tumor cell migration in ESCC via other pathways. CircNTRK2 altered NRIP1 function as miR-140-3p sponging, which helped promote the malignant biological cell behaviors (EMT included) of ESCC (97). Zhang et al. observed that hsa_circ_0012563 upregulation in ESCC could enhance cell migration and invasion by modulating the XRCC1/EMT axis. Furthermore, its expression is tightly linked to tumor pathogenesis and metastasis (98). Hsa_circ 0006948 can directly bind to miR-490-3p, which regulates ESCC progression by targeting the 3’-UTR of the oncogene HMGA2 (99). By binding to miR-124, circ_2646 facilitates ESCC cell proliferation, migration, invasion, and EMT (100). MiR-124 can slow ESCC development by suppressing PLP2 expression (100). Zhou et al. observed that circPDE3B functions as a ceRNA and promotes LAMA1-mediated EMT, metastasis, and proliferation in ESCC by acting as an miR-4766-5p sponge (101). Rescue experiments revealed that co-transfection of the circPDE3B vector or miR-4766-5p inhibitor partly reduced the inhibitory effect (101). Similarly, circARAP2 influences EMT and cancer stem cell differentiation by regulating miR-761/FOXM1 (102). Zhu et al. explained that circLONP2 facilitated ESCC proliferation and migration via miR-27b-3p sponging and governed its target gene—ZEB1—expression; consequently, circLONP2/miR-27b-3p-ZEB1 axis involvement might be an efficient strategy for ESCC treatment (103). CircFAM120B, also known as hsa_circ_0001666, is typically dysregulated in ESCC and is favorably correlated with overall survival (104). Essentially, circFAM120B significantly reduced the biological functions of ESCC by disrupting PKR to affect the p38/MAPK/EMT axis or sponging miR-661 to restore PPM1L expression (104).

The aforementioned data continuously verified the crucial role of circRNAs in malignant tumor invasion and metastasis, demonstrating that circRNAs are collectively involved in the EMT process by participating in modulating EMT-related pathways.

## 5 Clinical application of circRNA in ESCC

**Figure 3:**
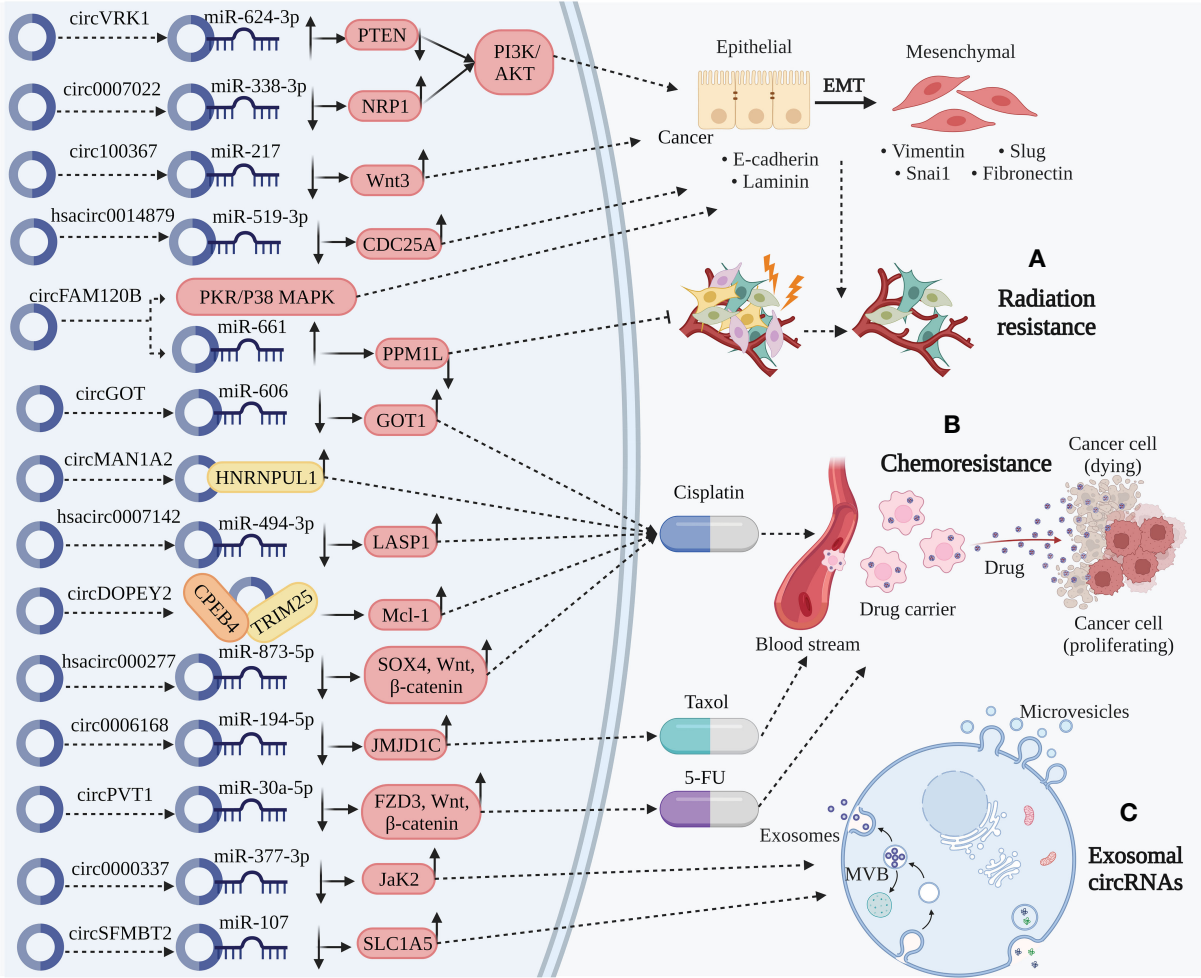
Clinical application of circRNA in ESCC. **(A)** Radiation resistance. CircVRK1 downregulate PETN by sponging miR-624-3p and inactivate the PI3K/AKT pathway and EMT. Circ_0007022 upregulate NRP1 by sponging miR-338-3p activating the PI3K/AKT pathway and EMT. Circ_100367 and hsa_circ_0014879 upregulate Wnt3 and CDC25A by sponging miR-217 and miR-519-3p, thereby promoting EMT. CircFAM120B significantly promote EMT by disrupting PKR to affect the p38/MAPK axis. Besides, circFAM120B downregulate PPM1L by sponging miR-661 directly functioning on radiation resistance. The role of circRNAs above participate in radiation resistance to affect the progression of ESCC. **(B)** Chemoresistance. CircMAN1A2 binding with HNRNPUL1 related to cisplatin resistance. CircDOPEY2 functions as a protein scaffold binding with CPEB4 and TRIM25, upregulating Mcl-1 translation and increasing cisplatin resistance. CircGOT1, hsa_circ_0007142, and hsa_circ_0000277 upregulate GOT1, LASP1, and SOX4/Wnt/β-catenin by sponging miR-606, miR-494-2p, and miR-873-5p increasing cisplatin resistance. Circ_0006168 upregulate JMJD1C by sponging miR-194-5p increasing Taxol resistance. CircPVT1 increase 5-FU resistance through miR-30a-5p/FZD3/Wnt/β-catenin axis. **(C)** Exosomal circRNAs. Circ_0000337 and circSFMBT2, serum exosomal circRNAs, upregulate JaK2 and SLC1A5 by sponging miR-377-3p and miR-107, thereby promoting carcinogenic activity of ESCC.

With the deepening of circRNA studies, various emerging targets have been identified and verified to be involved in the occurrence and development of ESCC (68, 192). Furthermore, current studies have revealed that circRNAs can be used as biomarkers. Due to their unique resistance to chemotherapy medications, targeted therapy can be carried out for patients with clinical chemotherapy resistance (**Figure 3**) (31, 193).

### 5.1 Regulating chemoradiation resistance in ESCC

Malignant tumors are often accompanied by metastasis postoperatively; therefore, radiotherapy and chemotherapy still need to be administered locally or systemically (194). However, due to the emergence of resistance, some people with cancer still experience local recurrence and lymph node metastases (195). Thus, the discovery of different targets to enhance treatments can be accomplished by understanding the regulatory processes of circRNAs implicated in radiation and chemotherapeutic resistance (196). The three major concerns associated with anticancer agents are drug resistance, metastasis, and EMT. According to available data, drug resistance and EMT are associated in many ways. Cancerous cells that are resistant to drugs might have higher EMT and invasive capabilities (197, 198). Accumulating evidence has clearly revealed that circRNAs play a significant role in EMT regulation and radiation resistance in ESCC. For instance, increased circVRK1 levels decreased the migration potential, reversed EMT progression, and drastically increased ESCC radiosensitivity by modulating the miR-624-3p/PETN axis and inactivating the PI3K/AKT signaling pathway (105). Zhang et al. verified that circ_0007022 can function as a miRNA sponge to regulate the miR-338-3p/NRP1 axis, activating EMT and the PI3K/AKT pathway to generate radiation resistance in ESCC (106). Circ_100367, an oncogenic circRNA in ESCC cells with a higher potency of EMT coordinated with miR-217, extraordinarily regulates the radioresistance of ESCC through the Wnt3 signaling pathway (107). Liu et al. demonstrated that hsa_circ_0014879 knockdown enhanced ESCC radiosensitivity and reduced ESCC migration and EMT via the miR-519-3p/CDC25A regulatory axis (108).

One of the most commonly used chemotherapeutic drugs is platinum, which is a complex barrier in cancer therapy (199). Albeit the mechanisms of platinum drug resistance have been the subject of numerous investigations, few efficient, targeted treatments are available (200–202). Mounting evidence suggests that circRNAs are involved in multiple cellular processes and malignancies, including ESCC (203, 204). Understanding the underlying causes of chemoresistance is crucial for developing effective ESCC treatments and preventing recurrence. Zhou et al. unveiled that circGOT1 functions as an oncogene in ESCC, stimulating cell proliferation, migration, glycolytic metabolism, and reducing apoptosis by acting as an miR-606 sponge to induce GOT1 expression (86). CircGOT1 knockdown restored the cisplatin resistant ESCC sensitivity to cisplatin (86). In patients with ESCC undergoing platinum-based chemotherapy postoperatively, increased HNRNPUL1 expression is linked to a poor prognosis with a greater likelihood of recurrence and shorter disease-free survival (DFS) (109). By controlling circMAN1A2 production, HNRNPUL1 inhibition reduces the cisplatin-chemoresistance of ESCCs (109). Chang et al. uncovered that circ_0007142 depletion can modulate the miR-494-3p/LASP1 axis, leading to cisplatin resistance in ESCC (110). Moreover, multiple chemotherapeutic drug resistance-associated proteins produce GST-π, which can reduce the cytotoxicity of chemotherapy medications (205). P-gp is an energy-dependent transporter that may combine with drugs and ATP to decrease medication concentration and supply energy for chemotherapeutic drug excretion (206). Remarkably, cisplatin resistant ESCCs with hsa_circ_0007142 silencing had lower P-gp and GST-π levels (110). Additionally, hsa_circ_0007142 knockdown significantly reduced cisplatin resistance in ESCC cells (110). Apoptosis is also closely associated with carcinogenesis. CircRNAs can tip the balance between cell death signaling and may improve chemoresistance efficacy (207). A BCL-2 family member that has been the subject of the most research is MCL-1, an anti-apoptotic oncoprotein (208). Mcl-1 is crucial for malignant biological functions because it mediates the apoptotic signaling pathway (209). It is also fundamentally altered, upregulated, and largely related to chemoresistance in several cancer (210). Liu et al. observed that in ESCC cells, circDOPEY2 functions as a protein scaffold to enhance CPEB4 ubiquitination and degradation in a TRIM25-dependent manner, increasing cisplatin-induced apoptosis by inhibiting CPEB4’s promotion of Mcl-1 translation and reducing cisplatin resistance (111). Another ESCC study revealed that hsa_circ_0000277 can modulate the miR-873-5p/SOX4/Wnt/β-catenin axis by acting as an miR-873-5p sponge to accelerate progression and chemoresistance in ESCC (112). JMJD1C facilitates tumor growth in various malignancies by promoting cancer cell growth and preventing apoptosis (211, 212). A study demonstrated that inhibiting circ_0006168 decreased tumor growth *in vivo* by sponging miR-194-5p and modulating JMJD1C, and *in vitro* by reducing cell proliferation, invasion, and migration, and Taxol-resistant ESCC apoptosis (113). Functionally, circPVT1 modulates the miR-30a-5p/FZD3/Wnt/β-catenin axis and ferroptosis, and circPVT1 knockdown can drastically increase the expression of ferroptosis-associated parameters MDA/Fe, which can improve chemoresistance in ESCC (114).

With accumulating evidence for circRNAs elucidated by research on chemoradiation resistance in ESCC as a new biomarker, circRNA has excellent potential in predicting the efficacy and prognosis of chemoradiotherapy or interfering with chemoradiation resistance and can be a target for clinical cancer therapy.

### 5.2 Exosomal circRNAs in ESCC TME

Exosomes are discoidal vesicles of 30–150 nm diameter that carry cargo derived from the host cell, such as proteins, lipids, DNA, and RNA. Exosomes carry their cargo to recipient cells, allowing immune and cancerous cells to communicate through them (213, 214). Cancer invasion and metastasis may be facilitated by exosome-mediated interactions between cancerous cells and the TME, encouraging EMT, angiogenesis, and immune escape (215). The TME is essential for intercellular information transmission and oncogenesis. Numerous studies have reported extensive and sustainable circRNAs in the exosomes (216–221). In a recent study, Liu et al. identified that serum exosomal hsa_circ_0026611 expression is markedly increased in ESCC, indicating that it can be utilized as a valuable indicator to distinguish lymph from non-lymph node-metastatic ESCC (115). Additionally, elevated hsa_circ_0026611 expression is strongly associated with poor overall survival (OS) and DFS (115). In another study, Zang et al. demonstrated that circ_0000337 was highly upregulated in ESCC (116). In addition, exosomes released by cisplatin-resistant ESCC cells that contain abundant circ_0000337 enhance the ability of ESCC cells to resist cisplatin and promote cell proliferation and metastasis (116). Xenotransplantation supported this hypothesis (116). Thus, exosomal circ_0000337 drastically affects cisplatin resistance in ESCC by modulating the miR-377-3p/Jak2 axis (116). Exosomal circSFMBT2 has been linked to a poor prognosis and is upregulated in ESCC (117). CircSFMBT2 silencing may slow cancer progression by preventing ESCC proliferation, invasion, and glutamine metabolism (117). Mechanistically, circSFMBT2 leads to SLC1A5 upregulation by sponging miR-107, thereby promoting carcinogenic activity (117). CircSFMBT2 exosomes may be an effective new therapeutic indicator for ESCC because they facilitate circSFMBT2 metastasis in ESCC cells, which causes the cells to exhibit the aforementioned malignant behaviors (117). Exo-circRNAs are defined by a transferrable target-specific capability, in addition to the initial physiological functions of circRNAs, since circRNAs are detected in exosomes (19). Continued studies may further reveal the involvement and promising effects of exo-circRNAs in the TME; thus, they require confirmation.

## 6 Discussion

As well as being affected by abnormal mutations in malignant cells, cancer is also associated with the composition of the TME. The TME is characterized by heterogeneous composition according to a large amount of research. Nevertheless, nearly all types of cancer can be treated with specific cells and mediators to improve patient health. There is increasing evidence that circRNAs play a crucial role in TME regulation. The exact physiological and pathological roles of circRNA in TME, as well as the underlying mechanisms, remain largely unknown. Currently, the interaction mechanisms between circRNAs and TME elements have received considerable attention. Establishing TME networks involving circRNAs may be an emerging direction for targeted therapies based on their interactions with circRNAs. Current findings on circRNAs have primarily concentrated on how they affect the bioactivity of malignancy, while systematic summaries for specific solid tumor microenvironments are lacking.

In this review, we discussed the biogenesis/degradation mechanisms and biological functions of circRNA occurrence. We systematically summarized the biological functions and clinical features of ESCC affected by circRNA dysregulation and its regulatory mechanisms in TME. Especially, in TME-related immune microenvironment, energy metabolism regulation, angiogenesis, hypoxia, epithelial-to-mesenchymal transition and tumor cell migration. Finally, we also discussed the mechanisms by which circRNAs drive chemoradiation resistance in ESCC and the role of the emerging exosomal circRNA in ESCC TME, thus providing insight into the future role of circRNA as a potential diagnostic indicator of ESCC. Nevertheless, there still has some relevant potential mechanisms that have not yet been elucidated in studies of circRNA in ESCC TME. In the immune microenvironment we focus on the relationship between circRNA and macrophage M2 polarization, the expression of other immune cells associated with TME such as T cells, NK cells, and the surface immune checkpoint molecules PD-L1/PD-1 in tumor cells, and the expression of important components of TME: stromal cells such as cancer-associated fibroblasts, endothelial cells, and pericytes, for which more studies are needed to explore their potential regulation. Researches on circRNA in ESCC TME regarding energy metabolism, angiogenesis, EMT, and hypoxia mechanisms is increasing and becoming more detailed. However, current studies on metabolic mechanisms are mainly focused on glycolysis, glutamine metabolism, proline metabolism, and oxidative phosphorylation. Other metabolic-related mechanisms, such as serine and lipid metabolism, need to be explored.

Clinical applications related to the role of circRNA in ESCC TME also need to be further explored, in addition to some of the above mechanistic studies to be elucidated. Despite the exploration of animal models to validate circRNA in chemoradiation resistance in ESCC, the applicability of circRNA-targeted therapy for ESCC in clinical trials remains to be investigated. Moreover, exosomal circRNAs play a crucial role in ESCC, providing new therapeutic targets and promising biomarkers for exosome-loaded small molecules that could be designed for ESCC diagnosis. We believe that with the investigation of circRNA function and mechanism in ESCC TME, it will definitely contribute to the developments of new strategies for ESCC treatment.

## Author contributions

JM, YS, and BZ designed and guided the review. JL wrote and edited the manuscript. HC helped with reference collection. All authors contributed to the article and approved the submitted version.
